# Orientation-Mediated Luminescence Enhancement and Spin-Orbit Coupling in ZnO Single Crystals

**DOI:** 10.3390/nano12132192

**Published:** 2022-06-26

**Authors:** Ali Hassan, Abbas Ahmad Khan, Yeong Hwan Ahn, Muhammad Azam, Muhammad Zubair, Wei Xue, Yu Cao

**Affiliations:** 1 China International Science & Technology Cooperation Base for Laser Processing Robotics, Wenzhou University, Wenzhou 325035, China; xm@wzu.edu.cn; 2 Zhejiang Provincial Key Laboratory of Laser Processing Robotics, College of Mechanical and Electrical Engineering, Wenzhou University, Wenzhou 325035, China; 3 Department of Physics and Department of Energy Systems Research, Ajou University, Suwon 16499, Korea; ahny@ajou.ac.kr; 4 Department of Physics, Faculty of Sciences, University of Central Punjab, Lahore 54000, Pakistan; muhammad.azam01@ucp.edu.pk; 5 Centre for Advanced Materials Application CEMEA, Slovak Academy of Sciences, Dubravska Cesta 9, 845 11 Bratislava, Slovakia; muhammad.zubair@savba.sk; 6 Oujiang Laboratory (Zhejiang Lab for Regenerative Medicine, Vision and Brain Health), Wenzhou University, Wenzhou 325000, China

**Keywords:** zinc oxide, single crystal, photoluminescence, exciton, impurities and defects

## Abstract

Temperature-, excitation wavelength-, and excitation power-dependent photoluminescence (PL) spectroscopy have been utilized to investigate the orientation-modulated near band edge emission (NBE) and deep level emission (DLE) of ZnO single crystals (SCs). The near-band-edge emission of ZnO SC with <0001> orientation exhibits strong and sharp emission intensity with suppressed deep level defects (mostly caused by oxygen vacancies V_o_). Furthermore, Raman analysis reveals that <0001> orientation has dominant E_2_ (high) and E_2_ (low) modes, indicating that this direction has better crystallinity. At low temperature, the neutral donor-to-bound exciton (D^o^X) transition dominates, regardless of the orientation, according to the temperature-dependent PL spectra. Moreover, free-exciton (FX) transition emerges at higher temperatures in all orientations. The PL intensity dependence on the excitation power has been described in terms of power-law (I~L^α^). Our results demonstrate that the α for <0001>, <1120>, and <1010> is (1.148), (1.180), and (1.184) respectively. In short, the comprehensive PL analysis suggests that D^o^X transitions are dominant in the NBE region, whereas oxygen vacancies (V_o_) are the dominant deep levels in ZnO. In addition, the <0001> orientation contains fewer V_o_-related defects with intense excitonic emission in the near band edge region than other counterparts, even at high temperature (~543 K). These results indicate that <0001> growth direction is favorable for fabricating ZnO-based highly efficient optoelectronic devices.

## 1. Introduction

Single crystals, especially from metal oxides, received enormous attention worldwide in the past few decades due to their distinguished optoelectronic and gas sensing properties over a large scale [1,2,3,4]. Single crystals of zinc oxide, a wide and direct bandgap (~3.37 eV) semiconductor, gained extra interest among other counterparts due to its unique luminescence feature at 378 nm wavelength and large exciton binding energy (60 meV) [5,6,7]. The intense near band edge emission and the large exciton binding energy make ZnO a promising candidate for optoelectronic devices such as laser diodes [8], light-emitting diodes [9], and photodetectors [10]. Moreover, the availability of polar and non-polar faces in zinc oxide single crystal adds variety in tuning its optical and electronic properties; for example, radiation absorption and emission from ultraviolet (UV) to near-infrared (NIR) region [11,12]. To achieve the best output from ZnO-based devices, it is important to select a suitable growth direction (polar and non-polar faces) depending upon own needs. For instance, Sasaki et al. [13] recently observed the large densities of surface states in O-faced ZnO single crystal which cause trap electrons near these surfaces and activate the ionized donor bound exciton (D^+^X) [14]. However, their results proposed that the Zn-faced single crystal has fewer surface states of ionized impurities, which results in the absence of an ionized donor bound exciton (D^+^X) peak. It is essential to control the non-recombination centers present in the single crystal to gain high device efficiency with low defect concentration [15,16,17].

The choice of different orientations depends on the desired property. Mostly, <0001> orientation has been found to be good for the growth of high UV emission ZnO-based devices (UV detectors or UV lasers). Recently, Jacobs et al. [18] reported the high near band edge emission with completely suppressed deep level defect luminescence in polar ZnO-based heterojunction light emitting diodes (LEDs). The suppression of deep levels enhances the device stability over a longer time and minimizes the heating effects produced by phonon vibrations. Valtiner et al. [3] examined the photo-corrosion mechanism of polar <0001> and non-polar <1010> ZnO single crystals. Their study shows the ideal stability at very low potential and under hydrogen evolution conditions on polar <0001> surface. In contrast, the non-polar <1010> surface was inactive for water splitting and sustained a higher degree of dissolution. Despite the considerable impact of polar and non-polar surfaces of ZnO single crystals on the modulation of luminescence and sensing properties, it has been noted that there are few reports presented in the literature to discuss their orientation-dependent photoluminescence mechanism; at room temperature as well as at low temperature [13,19,20].

Considering the above-mentioned facts, the present work is completely devoted to the understanding of luminescence mechanism and presence of different emission bands in polar <0001> and non-polar <1120>, <1010> ZnO single crystals from high temperature to low-temperature (543–123 K). Moreover, the effect of different excitation power on near band edge and deep level emission sites has also been discussed systematically. The current study will help to cognize the more detailed phenomena of luminescence characteristics and defect emission in different polar and non-polar ZnO single crystals, which will further pave the way to building more efficient optoelectronic devices. Despite having excellent photoelectric properties, the practical applications of ZnO-based optoelectronic devices have been restricted due to the unavailability of a defect-free homoepitaxial substrate that can minimize the lattice mismatches and ion diffusion offered by foreign substrates. Therefore, the current study proposed that the use of <0001> ZnO single crystal can solve the problem of lattice mismatch and ionic diffusion offered by a heteroepitaxial substrate. Furthermore, the heat mismatching or phonon generation can also be suppressed by the utilization of <0001> single crystal as a homoepitaxial substrate in ZnO-based optoelectronic devices. 

## 2. Materials and Methods

Hydrothermally grown as prepared ZnO single crystals, having dimensions 10 × 10 × 5 mm^3^ with three different orientations (<0001>, <1120>, <1010>) were purchased from (HF-Kejing, Hefei, China). An Andor 550 spectrometer (Oxford Instruments, Abingdon, UK) with two excitation wavelengths (320 nm, 405 nm) was employed to measure the micro-PL combined with a Newton 920 CCD detector (Oxford Instruments, Abingdon, United Kingdom). Micro-Raman for ZnO single crystals was measured using 405 nm and 532 nm wavelength in a backscattering mode of LAB Raman HR Raman spectrometer (Horiba Scientific, Kyoto, Japan). The excitation laser was focused on the sample using a confocal microscope having a 50× objective lens with a 5-micrometer spot size for 320 nm and 1-micrometer for 532 nm excitation wavelength. Here, it is worth mentioning that the spot size does not depend on the pump power of the excitation laser under confocal microscopic conditions. The X-ray diffraction (Rigaku D/MAX-2004 from Rigaku Corporation, Tokyo, Japan) was applied to characterize the structural analysis of single crystals. Field-emission electron microscopy (FESEM) (model Hitachi S-4800 from Hitachi, Ltd. Tokyo, Japan) equipped with the in situ energy dispersive X-ray spectroscopy (EDS) was implanted to check the morphology and elemental composition. X-ray photoelectron spectroscopy (XPS) from ThermoFisher Scientific, Waltham, MA, USA, model; Thermo Scientific K-Alpha was utilized to study the chemical bonding and core-level mapping of ZnO single crystals. The excitation source was Al Kα having photon energy of 1486.6 eV with a beam spot of 400 µm. The chamber pressure was kept 5 × 10^−7^ mBar with a working voltage of 12 kV and filament current of 6 mA. The conduction energy for core-level spectra was kept 50 eV with a step length of 0.1 eV. The binding energy of C 1 s (284.80 eV) was taken as a reference for this work. The atomic force microscopy (AFM) model (Bruker Dimension Icon, from Bruker Corporation, Billerica, MA, USA) has been used in tapping mode for surface topography and surface roughness study of single crystals.

## 3. Results and Discussions

### 3.1. Surface Morphology, X-ray Diffraction, and Raman Analysis

**Figure 1:**
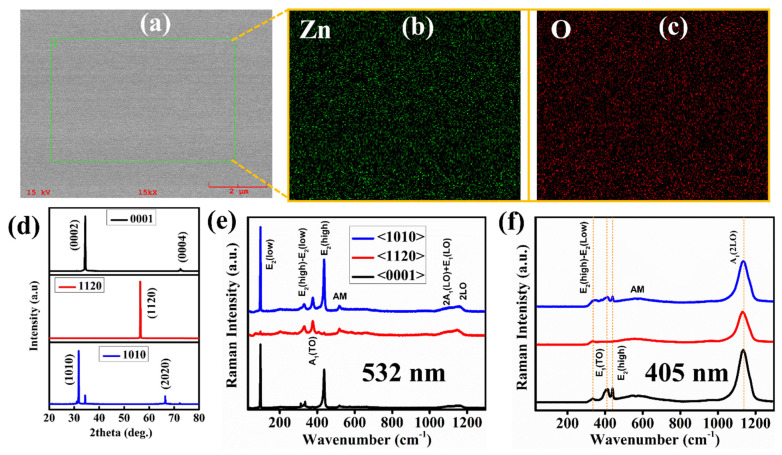
(**a**–**c**) Field-emission electron microscopy (FE-SEM) and energy dispersive spectroscopy (EDS) image of <0001> single crystal (**d**) XRD micrographs of all three ZnO single crystals <0001>, <1120> and <1010>. The Raman spectra of ZnO single crystals were measured with (**e**) an excitation wavelength of 532 nm and (**f**) 405 nm.

Figure 1a shows the smooth surface of as-received ZnO single crystals without any surface contamination (surface smoothness further verified via atomic force microscopic 2D image shown in Figure 4 discussed later). Moreover, the elemental analysis was carried out to check the sample’s elemental composition and it was found that the single crystals are Zn-rich with slightly high atomic percentage (at%, Zn~57.05, O~42.95) as shown in Figure 1b, c. Here, it is worth noting that the measured atomic percentage has been carried out by energy dispersive X-ray spectroscopy (EDS) which does not provide the elemental composition about whole single crystal as it only detects the surface. Moreover, the accuracy of EDS is affected by several other factors, including but not limited to the overlapping X-rays emission peaks, sample nature, and the X-ray escaping from the sample which did not reach the detector. The XRD graph (Figure 1d) exhibits that all three samples are grown in different preferential directions without any impurity peak. However, the <1010> orientation contains an additional peak (0002) at 2θ (34.3), which belongs to the <0001> orientation. It suggests that <1010> orientation of ZnO single crystal is not so stable. Franks et al. [11] also proposed that the <1010> orientation is unstable due to the higher surface O atoms attached per Zn tetrahedron. The peak (2020) is another crystallographic plane of <1010> orientation according to the JCPDS: 36-1451 of zinc oxide. The higher surface oxygen atoms in the <1010> can imbalance the stability of the <1010> orientation, which can cause the lattice mismatch in the <1010> orientation and some atoms which lie in the (0002) plane shows XRD reflection. Figure 1e,f shows the orientation-dependent Raman spectra measured at room temperature with two different excitation wavelengths. We have identified several characteristics vibrational modes for ZnO single crystals using 532 nm excitation wavelength, which corresponds to E_2_(low), E_2_(high)-E_2_(low), A_1_(TO), E_2_(high), AM, 2A_1_(LO)+E_1_(LO), and 2LO. However, the observed vibrational modes using 405 nm excitation wavelength are E_2_(high)-E_2_(low), E_1_(TO), E_2_(high), AM, and A_1_(2LO), respectively. Table 1 represents the corresponding wavenumbers associated with each Raman mode. It has been well noted that the ZnO single crystal contains a wurtzite structure that belongs to the P6_3_mc space group. The four atoms in the unit cell result in twelve phonon modes, of which nine are optical, and three are acoustic. The nine optical modes are further divided into the A_1_ branch, one double degenerate E_1_ branch, two double degenerate E_2_ branches, and two inactive B_1_ branches [21,22]. Here, A_1_ and E_1_ are predicted as the polar modes and further split into longitudinal optical (LO) and transverse optical (TO) phonons, respectively. The E_2_ mode contains the E_2_(high) and E_2_(low) optical modes; E_2_(high) mode arises as a result of vibration generated by the oxygen atom. On the other hand, E_2_(low) mode appears due to the vibration originated by the Zn sublattice [23].

Figure 1e shows that in <0001> and <1010>, the E_2_(low) and E_2_(high) modes are prominent, whereas in <1120> orientation, these modes are very weak, and A_1_(TO) is prominent. The appearance of A_1_(TO) is usually attributed to the oxygen vacancies or Zn interstitials or their complexes [24], this indicates that the oxygen vacancies or Zn interstitials are higher in this orientation. No shift has been observed in the main characteristic Raman peaks in all three orientations, which indicates that all single crystals are free from extrinsic defects or impurities. The only defects in these single crystals are intrinsic defects, which are either oxygen vacancies or Zn interstitials. Furthermore, the absence of A_1_(TO) in the <0001> orientation may be explained in terms of phonon Raman scattering intensity using lattice dynamics theory, which states that if the incident light is perpendicular to the <0001> direction, the A_1_(TO) vibration will not emerge. Figure 1f presents the Raman spectra of ZnO single crystals with different orientations measured at room temperature with 405 nm excitation wavelength. It has been discovered that regardless of the orientation, the excitation wavelength significantly impacts the vibrational characteristics of ZnO single crystals. It is clear that the dominating vibrational modes have shifted nominally when utilizing a 405 nm excitation wavelength. All three orientations contain the A_1_(2LO) dominant Raman modes with high intensity compared to other modes. The discovery of this phenomenon implies that changing the excitation wavelength produces precise energy that is necessary to activate specific modes. The electron-phonon coupling becomes robust when the excitation wavelength is comparable to the energy required to activate that specific Raman mode. For example, excitation at 405 nm provides enough energy to populate the transition from the A_1_(2LO) site. The 532 nm excitation, on the other hand, provides sufficient energy to ignite the optical transition from E_2_ modes. Furthermore, Cardona et al. proposed that if the excitation wavelength is just below the bandgap, the scattering efficiency of the 2LO process can be improved due to resonant events [26].

### 3.2. X-ray Photoelectron Spectroscopy Measurement

**Figure 2:**
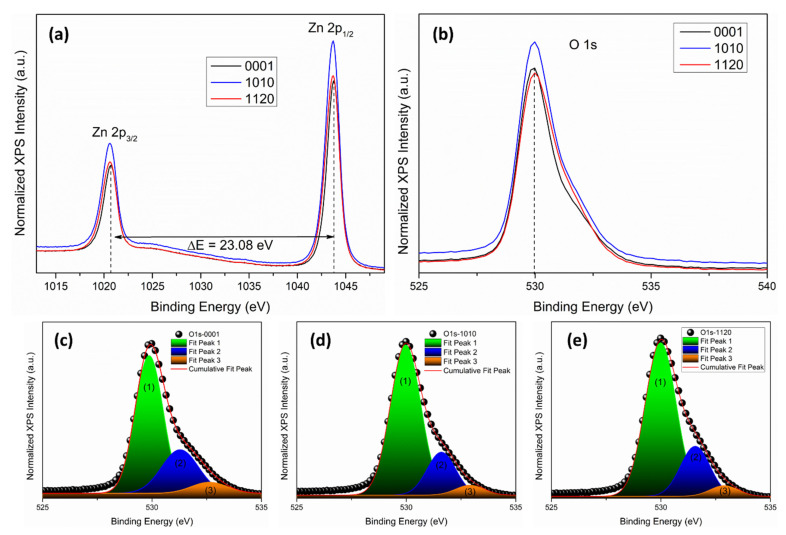
XPS core-level spectra of (**a**) Zn 2p and (**b**) O 1 s. The black dashed line indicates the peak position, and the horizontal double-head arrow represents the spin-orbit splitting (ΔE = 23.08 eV). Deconvoluted features of the O 1 s peak for (**c**) <0001>, (**d**) <1010>, and (**e**) <1120> orientation. Black spheres represent the experimental values, and the continuous red line indicates fitted results using the Gaussian fitting function.

**Figure 3:**
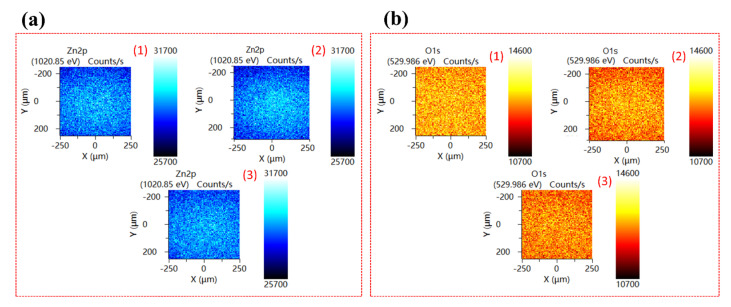
XPS core-level mapping of (**a**) Zn 2p and (**b**) O 1s ZnO single crystals; with polar (1) <0001>, and non-polar (2) <1010>, (3) <1120> orientations.

X-ray photoelectron spectroscopy (XPS) measurements were used to explore the bonding nature and chemical states of Zn and O atoms in single crystals. Figure 2a shows the Zn 2p core-level spectra of polar and non-polar ZnO single crystals. The binding energies of Zn 2p_3/2_ and Zn 2p_1/2_ peaks were observed at 1020.70 eV and 1043.78 eV for <0001>, 1020.62 eV and 1043.70 eV for <1010>, 1020.59 eV and 1043.67 eV for <1120> orientation, respectively. Spin-orbit splitting of ΔE~23.08 eV has been observed for all three samples, confirming that Zn’s chemical valence state at the surface of all three single crystals is Zn^2+^ oxidation state [27,28]. Moreover, a negative shift of 0.08 eV for <1010> and 0.11 eV for <1120> orientation has been observed in the binding energies of Zn 2p core-level spectra, which indicates that the majority of Zn atoms in the tetrahedral wurtzite geometry are in Zn^2+^ oxidation state. However, the presence of Zn_i_ and V_Zn_ cannot be ruled out [29]. Furthermore, a shift to the lower binding energy of Zn 2p_3/2_ for <1010> and <1120> orientations indicates that the oxygen vacancies present within the bulk crystal lattice dominate and could be a reason for green luminescence in ZnO (discussed in room-temperature PL section). The normalized core-level spectra of O 1 s for ZnO single crystals with three distinct orientations are shown in Figure 2b. The asymmetric structure of the O 1 s peak is due to the presence of different oxygen coordination in all three orientations [30]. To get a better qualitative analysis of different oxygen coordination and their concentration, we deconvoluted the O 1 s spectra using the Gaussian fitting function (Figure 2c–e). The three Gaussian peaks at ~529.86 ± 0.12 eV (Peak 1), ~531.28 ± 0.12 eV (Peak 2), and 532.61 ± 0.12 eV (Peak 3) were observed in all three orientations of the ZnO single crystal. All peaks show a shift toward high binding energy in non-polar orientation, which could be attributed to the surface oxygen vacancies of ZnO as observed by Tay et al. [29]. Here, peak-1 represents the O^2-^ ions in Zn-O bonding of the wurtzite structure of ZnO, peak-2 attributed to the oxygen vacancies within the crystal lattice of ZnO, whereas peak-3 denotes the loosely bounded oxygen atoms on the surface of ZnO single crystals, which belongs to some specific species like adsorbed O_2_ and H_2_O, or −CO_3_ [31]. We integrated the total area of O 1 s peak and the ratio of each deconvoluted peak and found that the ratio of peak-1 and peak-2 in <1010> and <1120> is higher than that of <0001> orientation, which indicates that the oxygen vacancies/defects and O^2+^ ions within the crystal lattice in these two orientations are present in excess. XPS core-level mapping of Zn 2p and O 1 s (Figure 3a,b) also confirms that the oxygen-related defects at the surface of <1010> and <1120> non-polar ZnO single crystal are quite high as compared to the polar <0001> ZnO single crystal.

### 3.3. Atomic Force Microscopy Analysis

**Figure 4:**
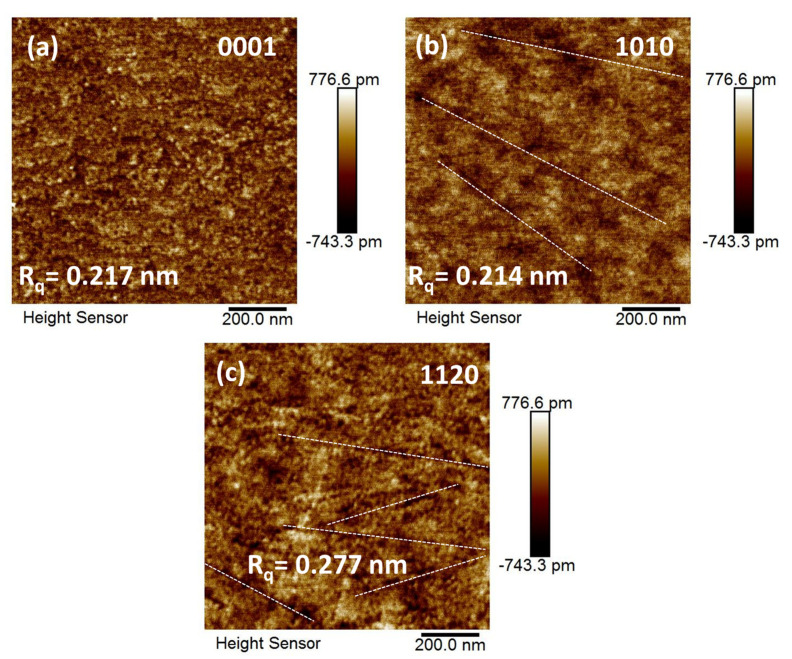
Two-dimensional AFM images of polar (**a**) <0001> and non-polar (**b**) <1010>, (**c**) <1120> ZnO single crystals. The white dashed line indicates the nano-scale cracks on the surface of single crystals. The surface roughness value of each single crystal surface was measured with a minimal standard deviation of 0.035 nm.

To deeply investigate the surface morphology and roughness of ZnO single crystals, atomic force microscopic (AFM) analysis in tapping mode has been performed, as shown in Figure 4a–c. The 2D micrographs indicate that surface of all three single crystals is relatively smooth with a surface roughness ranging from 0.214 nm to 0.277 nm and with a height (Z) sensor noise level or error of 0.035 nm, while a few cracks (white dashed lines) occur at the surface of <1010> and <1120> non-polar orientation which could be formed after synthesis and can be neglected. These results indicate that the morphology-related effects (grain size and shape) are not dominant in modulating the optoelectronic properties of ZnO single crystals in the present situation. Moreover, it is well-known that the shape and size of grain have an insignificant dependence on the polarity of a single crystal, if there is no substantial doping or post-treatment effect taken place. In contrast, the preparation route, post-annealing treatment, and doping can tailor the shape and size of grain and consequently modulate the surface roughness [32,33].

### 3.4. Room-Temperature and Low-Temperature Photoluminescence Spectroscopy

**Figure 5:**
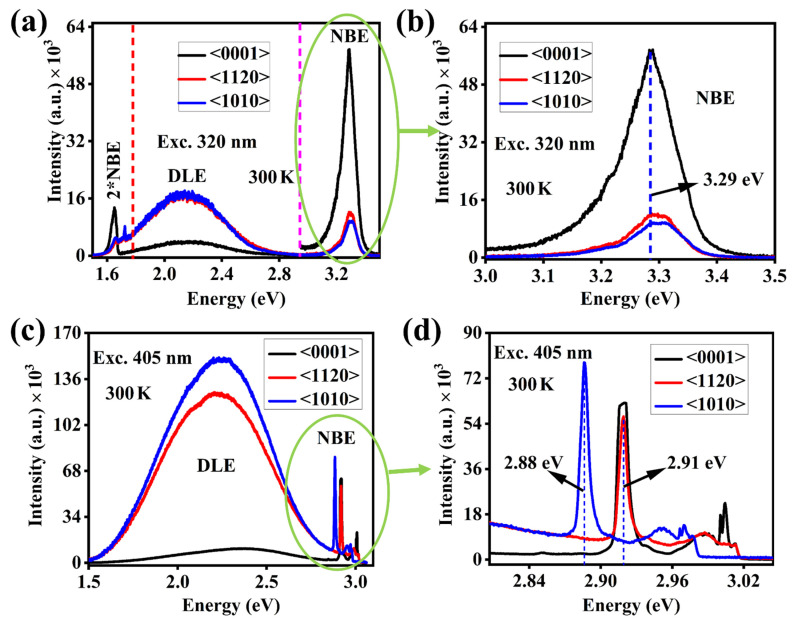
Room-temperature full scan PL spectra of ZnO single crystal with three different orientations (**a**,**b**) measured with 320 nm excitation laser, (**c**,**d**) measured using 405 nm excitation laser. Here NBE and DLE represents the near-band-edge and deep level emission, respectively.

**Figure 6:**
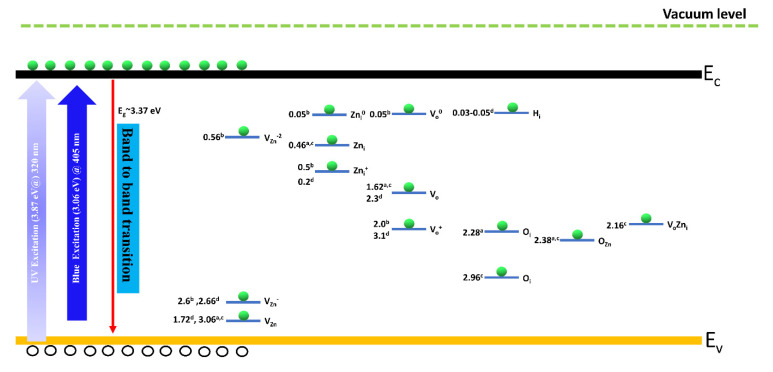
Schematic illustration of energy levels of different possible intrinsic defects in ZnO. The values has been extracted from previous reports: (a) ref. [38] (b) ref. [39] (c) ref. [40] and (d) ref. [41]. The light blue and bluish violet arrow represent the excitation wavelength used in the current study, V_Zn_, V_Zn_^−^, and V_Zn_^−2^ indicate the neutral, singly charged, and doubly charged Zn vacancies, respectively. Neutral and singly charged Zn interstitials are denoted by Zn_i_, and Zn_i_^+^. Neutral and singly charged oxygen vacancies are denoted by V_o_, V_o_^0^, and V_o_^+^. O_i_ and H_i_ indicate the oxygen and hydrogen interstitials, respectively. The V_o_Zn_i_ here represents the defect complex containing the oxygen vacancy and Zn interstitial.

The room-temperature (300 K) photoluminescence measurements have been performed to examine the various emissions sites (from near band edge to deep levels emission) dependency on the orientation of single crystals. Figure 5a,b shows that the highest near band edge emission with low emission from deep levels has been obtained from <0001> sample when the samples were excited with the excitation wavelength 320 nm, and the other two SCs gave very low emission from near band edge and high emission from deep levels. Even the excitation wavelength is comparable with the bandgap of the ZnO but different intensity from different orientation points that the emission sites concentration varies as we change the orientation. The presence of high NBE emission in <0001> face with weak emission in DLE region states that, in <0001> the electron-hole recombination is dominant and the vacancies level is lower in this orientation as we know that the deep level emission is mainly attributed to various intrinsic defects as shown in Figure 6 (such as V_Zn_, V_O_, Zn_i_, and O_i_) [34,35,36]. Generally, in intrinsic ZnO, V_o_ and Zn_i_ are two notable defects that act as deep and shallow defects, respectively [37]. The difference in defect-luminescence from ZnO with different orientations can be described based on the formation energies of these defects. 

Recently, some theoretical studies [34,42,43] suggested that the electronic formation energy of V_o_ is (0.51 eV) smaller than the formation energy of Zn_i_. The V_o_ formation energy in the orientations <1120> and <1010> will most likely be slightly lower than in the orientation <0001>, making it easier to populate these deep levels with more charge carriers. As a result, the DLE region produce more transitions, and the luminescence intensity automatically increases. We also employed a 405 nm excitation laser to measure the PL spectra of ZnO single crystals to investigate the effect of excitation wavelength on emission intensity. As demonstrated in Figure 5c, the 405 nm wavelength stimulated more transitions from the deep levels while emitting negligibly from the near band edge area. This behavior is reasonable since the 405 nm excitation wavelength does not transmit enough energy to activate the electron present in the conduction bands (3.37 eV). However, the transferred energy is enough to excite the deep levels. Furthermore, even with the 405 nm excitation, the <0001> orientation emits weakly in the DLE area, which indicates that the emission from deep levels depends not only on the excitation wavelength but also on the single crystal orientation, which may be because the deep levels sites present in ZnO single crystals are intrinsic in nature. However, the <0001> orientation may not be favorable for the presence of these intrinsic defect sites. The NBE region excited with 405 nm wavelength shows the sharp emission at slightly different emission energy (2.91 eV for <0001> and <1120> orientation whereas 2.88 eV for <1010> orientation) plus some background emission in this region (Figure 5d) due to the scattering of an incident photon with the electron presents at the conduction band minima. These peaks could be the 2LO Raman scattering. Figure 2b,d shows a modest blueshift in NBE emission with a higher excitation wavelength, indicating that oxygen vacancies play a significant role in this orientation, since the blueshift without any external doping is attributable to the presence of intrinsic oxygen-related defects [44,45,46].

**Figure 7:**
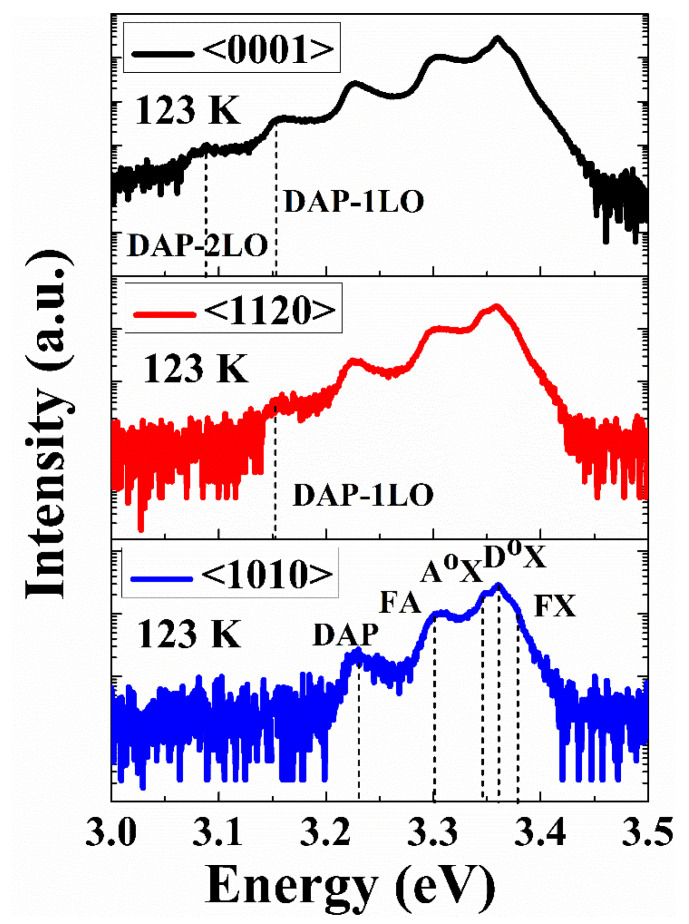
Low-temperature PL spectra of ZnO single crystals having three different orientations.

For better understanding of different emission centers, present in the NBE region, we have investigated the PL spectra of all three orientations at 123 K, as shown in Figure 7. All three single crystals show five dominant peaks at 3.374, 3.360, 3.346, 3.303, and 3.227 eV, representing the free exciton transition (FX), neutral donor-bound exciton (D^o^X) peak, neutral acceptor-bound exciton (A^o^X) peak, free electron to acceptor transition (FA) peak, and donor to acceptor pair transition (DAP) peak respectively. However, the <0001> orientation contains two additional peaks at 3.154 eV and 3.083 eV, which are recognized as the first-order longitudinal optical phonon replica of DAP transition DAP-1LO and DAP-2LO respectively. Conversely, the <1120> spectra only contain the DAP-1LO peak. The energy separation between these LO phonon replicas was found to be (hω_LO_ = 70–73 meV), consistent with previous reports [47,48]. Semiconductor such as ZnO is well-known for its highly polar nature, making it significant for exciton-phonon coupling. However, the bound excitons are supposed to be the extrinsic transitions that originate from dopants or defects and create discrete energy states for the charge carriers in the bandgap region. In the present case, we used pure ZnO single crystals without any extrinsic dopant, meaning that the transitions from the bound excitons result from intrinsic defects. Based on previous theoretical predictions, these defects could be either V_o_ or Zn_i_ [49,50,51]. 

**Figure 8:**
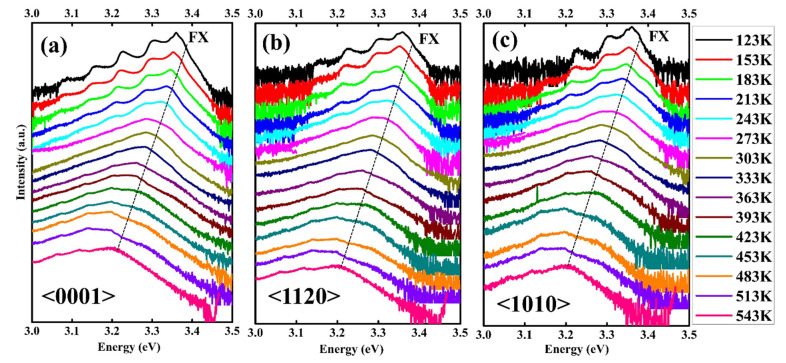
Temperature-dependent near-band-edge emission PL spectra of ZnO Single crystals with (**a**) <0001> (**b**) <1120> and (**c**) <1010> orientation measured over the temperature range 123–543 K using 320 nm excitation laser. The free-exciton (FX) transition dominates at elevated temperature.

Figure 8a–c represents the temperature-dependent photoluminescence spectra from cryogenic temperature (123 K) to slight high temperature (543 K) for orientation <0001>, <1120>, and <1010> respectively in the NBE region (3.0–3.5 eV). The PL spectra contain several emission centers, the position and intensity of which varies with the temperature. The neutral donor-bound exciton peak (D^o^X) dominates at low temperatures in all three orientations. However, the FX transition becomes more prominent as the temperature increases due to small localization energy [52]. Additionally, the DAP emission shows a redshift with an increase in the temperature. The energy of a photon resulting from the radiative recombination of a DAP transition is given by the following relation,

(1) hν=Eg−EA−ED+q2εr

**Figure 9:**
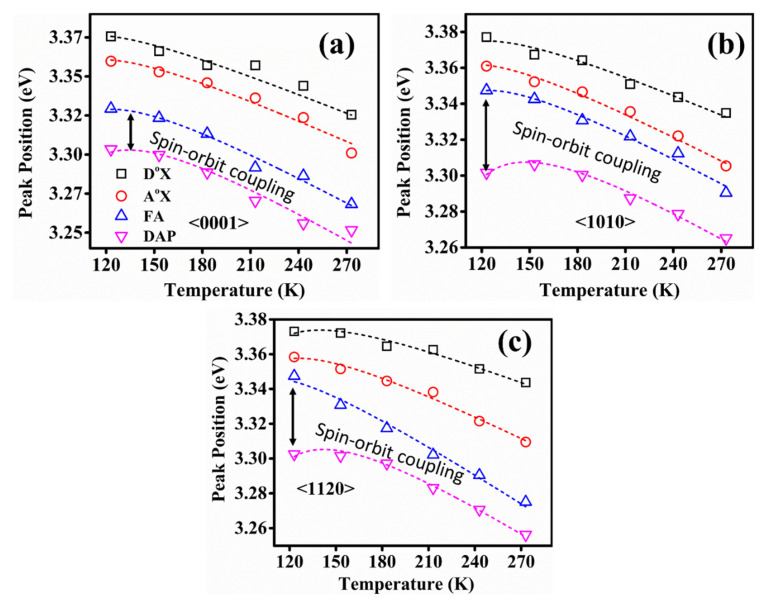
Temperature-dependent variation of peak positions for D^o^X, A^o^X, FA, and DAP transitions of (**a**) <0001> (**b**) <1120> and (**c**) <1010> orientations, respectively. Symbols indicate the experimentally observed peak positions of each transition and dashed lines are the fitting of experimental values with Varshni’s empirical formula.

Here, *E_g_* represents the bandgap energy, *E_A_* and *E_D_* are acceptor and donor binding energies, respectively, *ɛ* is the dielectric constant, and “*r*” is the distance between acceptor and donor. While increasing the temperature, the carriers on the DAP site with smaller distance are released into the band and as a result, the line is shifted toward low energy. Moreover, the neutral donor to bound exciton transition (D^o^X) shows a blueshift with the increase in temperature since at elevated temperatures; the localized carriers occupy higher energy states after getting thermalized. We analyzed the temperature dependence of FX, D^o^X, A^o^X, FA, and DAP emission centers for all three single crystals using Varshni’s formula, as shown in Figure 9a–c [53]. According to this relation,

(2) Eg(T)=Eg(0)−αT2(β+T)

Here, *E_g_*(0) is the bandgap energy at *T* = 0, *T* is the absolute temperature whereas *α* and *β* are specific constants for ZnO. The obtained values of these constants for three orientation are *E_g_*(0) ~3.37 eV; *α* ~1.04, 1.02, 1.09 meV/K; *β* ~897, 928, and 1016 K for <0001>, <1120>, and <1010> respectively. Varshni’s formula gives the best fit to all the five emission centers for single crystals, and the values obtained in (Table S1) are in good agreement with previous results, except a slight deviation which is negligible as the experimental conditions are not the same as Rai et al. [54] also reported a deviation by a factor of 6 from Giles et al. [55] where the sample preparation route of both reports is totally different. Therefore, we stated that the slight deviation is ignorable due to the change in experimental conditions [56,57,58]. Furthermore, in all samples, the peaks represent the linearly decreasing trend, which means that the peak position slightly shifts toward the lower energy with increased temperature, this is quite understandable, as we know that the increase in temperature provides extra energy to the crystal lattice in the form of thermal vibrations, which lowers the binding energies of these charged or neutral pairs presented on these particular sites. As a result, the transition from these sites becomes possible with an excited photon even with slightly less energy. Moreover, the spin-orbit coupling between free exciton and donor to acceptor pair has been found to be 20 meV for polar <0001> and 40 meV for non-polar <1010>, and <1120> orientation, which shows a decreasing trend with an increase in temperature. The observed results are in good agreement with previously published reports [59] as the extraction of spin-orbit coupling from the excitonic peak position is quite well-established. There are several studies which theoretically estimated the spin-orbit coupling in ZnO using density functional theory and they reported a value between 16 and 20 meV, for example the work done by Walter et al. [59] which suggested that the value of spin-orbit coupling in ZnO is 20 meV. D.C Look et al. [60] also extracted the spin-orbit coupling for ZnO from PL spectra where they stated that the value is 16 meV. Besides ZnO, Christopher et al. [61] adopted the same strategy to extract the spin-orbit coupling of MoS_2_ monolayers just by plotting the peak position over a range of temperature. Considering these all, we think that our adopted technique is a well-established strategy to extract the spin-orbit coupling for our ZnO which even provided the coupling value close to the published literature. Furthermore, the cause of a slight increase in spin-orbit coupling with change in polarity has not been discussed elsewhere. We postulate that it could be the lattice position of Zn atoms in the ZnO crystal lattice, which mediates the coupling between electronic spin and its orbital motion around the nucleus. The electrons of interstitial Zn atoms may face a repulsive interaction with other electrons of neighboring atoms, as the distance between interstitial position and substitutional position is comparatively small. This repulsive force from the surrounding may cause a slight increase in spin-orbit coupling in non-polar ZnO single crystals as they are defect-rich orientations confirmed by XPS and room-temperature photoluminescence analysis.

Figure S1 in Supplementary Materials show the complete scan temperature-dependent PL spectra of ZnO single crystals having three different orientations. Emission centers in the NBE and DLE regions have been found in all orientations with a difference in intensities and full-width half maxima. Moreover, one additional peak (2*NBE) has also been noted at ~1.65 eV, which is a replica of NBE, and it will disappear if we use a long pass filter to capture DLE peak only. The ratio of deep levels emission to the near band edge emission intensity has been calculated for three ZnO single crystals and shown in Table S1 in the Supplementary Materials. It has been noted that the single crystal with orientation <1010> possesses a higher ratio of I_DLE_/I_NBE_ (1.76), and the single crystal with <0001> orientation represents the lowest ratio of (0.04), this indicates that the <0001> orientation contains fewer emission sites in the deep level regions, which means that the crystal grown in this direction is more suitable for UV optical emission. Moreover, it has also been observed that the deep level emission sites are more prevalent in the <1120> and <1010> orientations with decreasing temperature. However, in the <0001> orientation, the effect of these defect-related emission sites is still ignorable even at lower temperatures, and the near band edge emission is still dominant in this orientation. It is likely that, in <0001> orientation, the deep level emission sites lie at higher energy levels indicating that the exciting photon does not have enough energy to activate these luminescent centers (Figure 6) or the ZnO single crystal has significantly less defect densities in this orientation.

### 3.5. Excitation-Wavelength-Dependent Photoluminescence Spectroscopy

**Figure 10:**
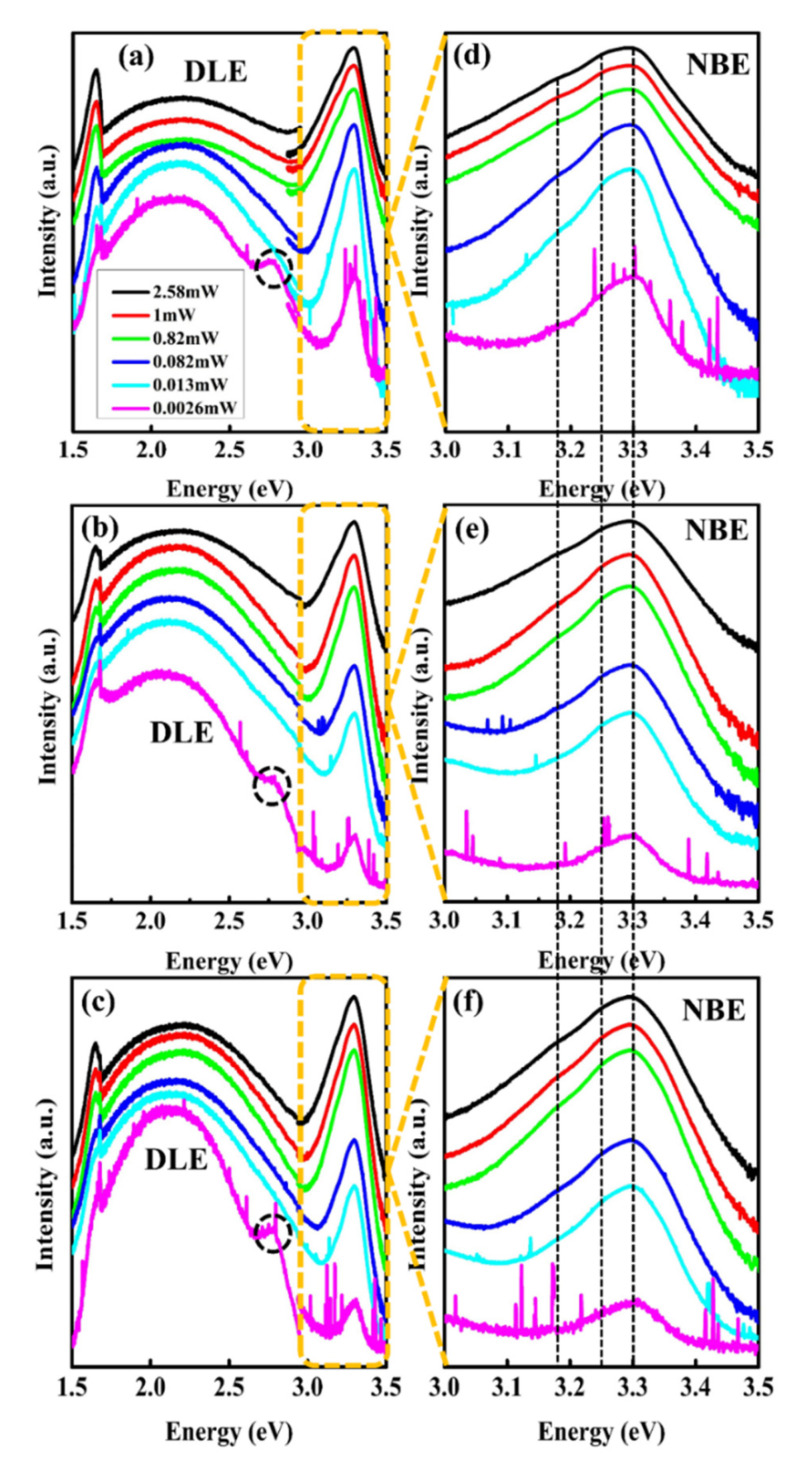
Excitation power-dependent photoluminescence spectra of (**a**) <0001> (**b**) <1120> and (**c**) <1010> orientation of ZnO single crystal. The dashed circle is an “unknown emission band” which is not related to the ZnO but could be scattering from system itself. Figure (**d**–**f**) represents the zoom-in on near band edge emission region of <0001>, <1120>, and <1010> orientation, respectively. Here NBE and DLE represent the near-band-edge and deep level emission, respectively.

Figure 10a–c exhibits the excitation power-dependent photoluminescence spectra of ZnO single crystals. We have used the low excitation power of 2.6 μW to the highest power of 2.58 mW. It has been noted that the highest photoluminescence in NBE region has been achieved with all orientations using 2.58 mW. As the excitation power decreases, the NBE peaks intensity also decreases whereas the deep level emission dominates at low excitation power, which indicates that the NBE peak intensity has a directly proportional relationship with the excitation power (the relationship of calculated peak position, peak intensity, FWHM, and the ratio of NBE/DLE as a function of excitation power has been elucidated in the Figure S2 in the Supplementary Materials), this is very unpretentious, as the excitation power provides the specific energy to excite the charge carriers present in the valence band. Every electron excited by the incident photon must absorb that specific energy (~equal to the bandgap of that semiconductor material) before transfer to the conduction band. If the excitation power is low, the incident photon will not transfer much energy required to excite the electrons to the conduction band, then there will be no band-to-band transition; however emission from low energy levels or defect sites still has the possibility. Therefore, the emission from DLE dominates at lower excitation power as shown in Figure 10a–c. Similarly, if we use the excitation power that has energy greater than the bandgap energy of that material, in this case, band-to-band transition will be suppressed due to phonon bottleneck effect. As a result, phonon generation suppresses the electron-hole pair recombination and low NBE emission will be observed. Therefore, it is essential to use the excitation power which can transfer the photon energy equal to the bandgap of that material [62]. Moreover, it has been noted that the same excitation power used for different orientations of ZnO single crystal gives different intensities of luminescence bands present in the ZnO. It means that these luminescence centers are from the intrinsic defect states of ZnO, which does not influence external source. Furthermore, an emission band (at 2.78 eV) emerge in all orientations at low excitation power (0.0026 mW) which is not related to the ZnO sample. We suspect that this emission could be scattering from the system itself as there is no evidence of emission band in ZnO in the literature and it is also not present in other spectra while using higher excitation power. Therefore, we call it “unknown emission” band.

**Figure 11:**
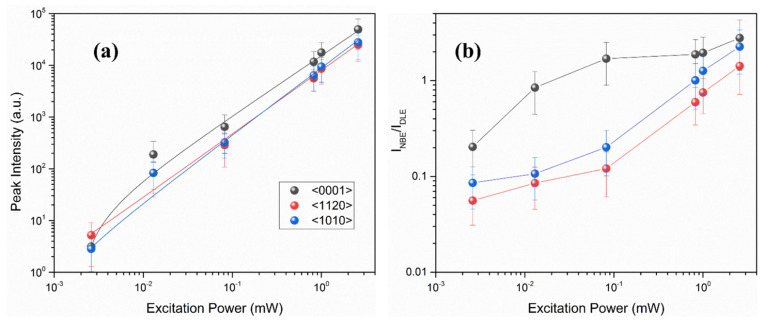
Excitation power-dependent (**a**) peak intensity of D^o^X, dashed lines are the fitting of experimental data using power law (**b**) the ratio of near band edge emission intensity to the deep level emission intensity for ZnO single crystal having different orientation. The peak intensity values used here have been extracted from Figure 10 without further processing.

Figure 10d–f represents the NBE region of all three orientations. It is well noted that the FX and D^o^X merge after 1 mW excitation power for all orientations. The D^o^X transition becomes dominant at low excitation power. However, as the excitation power increases, more and more DAPs are excited, contributing to the final emission; this happens as a result of the decreased distance between the donor and acceptor, which increases the Coulomb energy in Equation (1) and leads to the higher DAP peak energy. To further evaluate the D^o^X transition characteristics, we depicted the integrated PL intensities of D^o^X for three orientations of ZnO as a function of excitation power measured at room temperature, as shown in Figure 11a. It is worth noting that the PL intensity (*I*) increases with the excitation power (*L*), following the below relation,

(3) $$I=\eta L^{\alpha }$$

Here, *η* is the emission efficiency, and *α* represents the radiative recombination mechanism. For the excitation light having the energy higher than the bandgap energy of the target material, usually 1 < *α* < 2 for exciton-like transitions and *α* < 1 for free-to-bound and donor-to-acceptor like transitions [63,64]. The above relation is extensively used as a shred of evidence for finding the origin of the UV emission. In the present work, *α* has been observed to be 1.148±0.0892, 1.180±0.0950, and 1.184±0.0770 for <0001>, <1120>, and <1010> orientations, respectively, which makes it evident that exciton-like transitions are dominant in these samples. A distinct peak appears at 2.78 eV in all three orientations when we used the lowest excitation power (0.0026 mW), while it disappears at other excitation power conditions. This peak is not related to the ZnO samples but rather to the system. Moreover, we have also examined the emission intensity of the NBE peak and DLE peak as a function of excitation power, as shown in Figure 11b. It has been observed that <0001> contains a higher ratio at low excitation power. However, at higher excitation power the difference is not so significant. The trend suggests that the deep levels present in <1120> and <1010> orientations are intrinsic, possibly the oxygen vacancies (V_o_). The density of these defects is lower in <0001> orientation, giving rise to a high NBE/DLE ratio even at low excitation power. In short, the current results elucidate that the development of defect-free homoepitaxial substrate for ZnO-based optoelectronic devices, the <0001> orientation will be much beneficial as the fraction of intrinsic oxygen-related defects is less. Furthermore, the free-excitonic transitions are dominant at higher temperatures in ZnO which can be helpful in the fabrication of ZnO-based UV diodes.

## 4. Conclusions

A detailed investigation of temperature-dependent, excitation power-dependent, and orientation-mediated photoluminescence of ZnO single crystals has been carried out systematically. The room-temperature PL spectra of ZnO single crystal with different excitation wavelengths indicate that the excitation wavelength comparable to or slightly below the bandgap energy, causing more transition in near band edge area with less deep level transition indicating that the choice of excitation wavelength can suppress the emission from deep levels which is required to fabricate ZnO-based high-efficient UV diodes. However, even utilizing the same excitation wavelength, the orientation of a single crystal is equally critical for achieving intense NBE emission. In our case, we discovered that the <0001> orientation is better in terms of high emission in NBE with low DLE emission which is required for the development of homoepitaxial substrate for ZnO-based optoelectronic devices. The temperature-dependent PL measurements show multiple emission centers contributing to the near band edge emission region with dominant behavior of D^o^X transition in all samples at low temperature (123 K). The FX transition became more obvious as the temperature increased (543 K), indicating the free-excitonic transitions are dominant at higher temperature in ZnO, which can be helpful in fabricating ZnO-based UV diodes. The excitation power-dependent Photoluminescence results suggest that the <0001> orientation is more suitable for high excitonic emission with dominant D^o^X transition. Furthermore, we discovered that oxygen vacancies are the primary source of deep level emission in ZnO, and the larger densities of these inherent defects are present in <1120> and <1010> orientations. Therefore, the current study proposed that the use of <0001> ZnO single crystal can solve the problem of lattice mismatch and ionic diffusion offered by the heteroepitaxial substrate. Furthermore, the heat mismatching or phonon generation can also be suppressed by utilizing <0001> single crystal as a homoepitaxial substrate for ZnO-based optoelectronic devices.

## Figures and Tables

**Table 1:** Summary of Raman vibrational modes in the literature and observed in current study for ZnO single crystals with polar and non-polar faces.

Sr#	Raman Shift (cm^−1^) of ZnO Single Crystals Measured in the Current Study	Raman Shift (cm^−1^) of ZnO Single Crystals Reported in the Literature [24,25]	Peak Identity	Symmetry
1	97	99	E_2_(low)	E_2_ (Brillouin zone Γ)
2	331	333	E_2_(high)−E_2_(low)	E_2_, E_1_ (Brillouin zone Γ)
3	375	379	A_1_(TO)	A_1_ (Brillouin zone Γ)
4	435	438	E_2_(high)	E_2_ (Brillouin zone Γ)
5	518		AM	-
6	1097	1102	2A_1_(LO) + E_1_(LO)	A_1_, E_1_
7	1136	1138	A_1_, 2LO	-
8	1159	1154	2LO	Wurtzite ZnO, 2LO

## Data Availability

The data are contained within the article or Supplementary Materials.

## Supplementary Materials

The following supporting information can be downloaded at: https://www.mdpi.com/article/10.3390/nano12132192/s1, Figure S1: Complete scan of temperature-dependent photoluminescence spectra of (a) <0001> (b) <1120> (c) <1010> orientation of ZnO single crystals measured with an excitation wavelength of 320 nm from the temperature range 123–543 K. Figure S2: For ZnO single crystals with three distinct orientations, the relationship between (a) peak intensity (b) peak position (c) FWHM (d) ratio of NBE/DLE intensity as a function of excitation power is shown. The dashed lines represent a linear fit of the experimentally observed data points, while the symbols represent experimentally observed data. Table S1: Room-temperature PL peaks position, intensity ratio, and the Varshni’s fit parameters for ZnO single crystals with different orientations.
