# Supplementary material for: Orientation-Mediated Luminescence Enhancement and Spin-Orbit Coupling in ZnO Single Crystals
Source: Nanomaterials (Basel). 2022 Jun 26;12(13):2192. doi: 10.3390/nano12132192 (PMC9268195; doi:10.3390/nano12132192)
Supplement: Supplementary file 1 [file nanomaterials-12-02192-s001.zip › nanomaterials-1763990-supplementary.pdf]

Supplementary Materials

Orientation-Mediated Luminescence Enhancement and Spin-Orbit Coupling in ZnO Single Crystals

Ali Hassan [1,2,*], Abbas Ahmad Khan [3,*], Yeong Hwan Ahn [3], Muhammad Azam [4], Muhammad Zubair [5], Wei Xue [1,6] and Yu Cao [1,6,*]

1 China International Science & Technology Cooperation Base for Laser Processing Robotics, Wenzhou University, Wenzhou 325035, China; xm@wzu.edu.cn
2 Zhejiang Provincial Key Laboratory of Laser Processing Robotics, College of Mechanical and Electrical Engineering, Wenzhou University, Wenzhou 325035, China
3 Department of Physics and Department of Energy Systems Research, Ajou University, Suwon 16499, Korea; ahny@ajou.ac.kr
4 Department of Physics, Faculty of Sciences, University of Central Punjab, Lahore 54000, Pakistan; muhammad.azam01@ucp.edu.pk
5 Centre for Advanced Materials Application CEMEA, Slovak Academy of Sciences, Dubravska Cesta 9, 845 11 Bratislava, Slovak Republic; muhammad.zubair@savba.sk
6 Oujiang Laboratory (Zhejiang Lab for Regenerative Medicine, Vision and Brain Health), Wenzhou University, Wenzhou 325000, China
* Correspondence: alirao@wzu.edu.cn (A.H.); abbaskhan@ajou.ac.kr (A.A.K.); yucao@wzu.edu.cn (Y.C.)

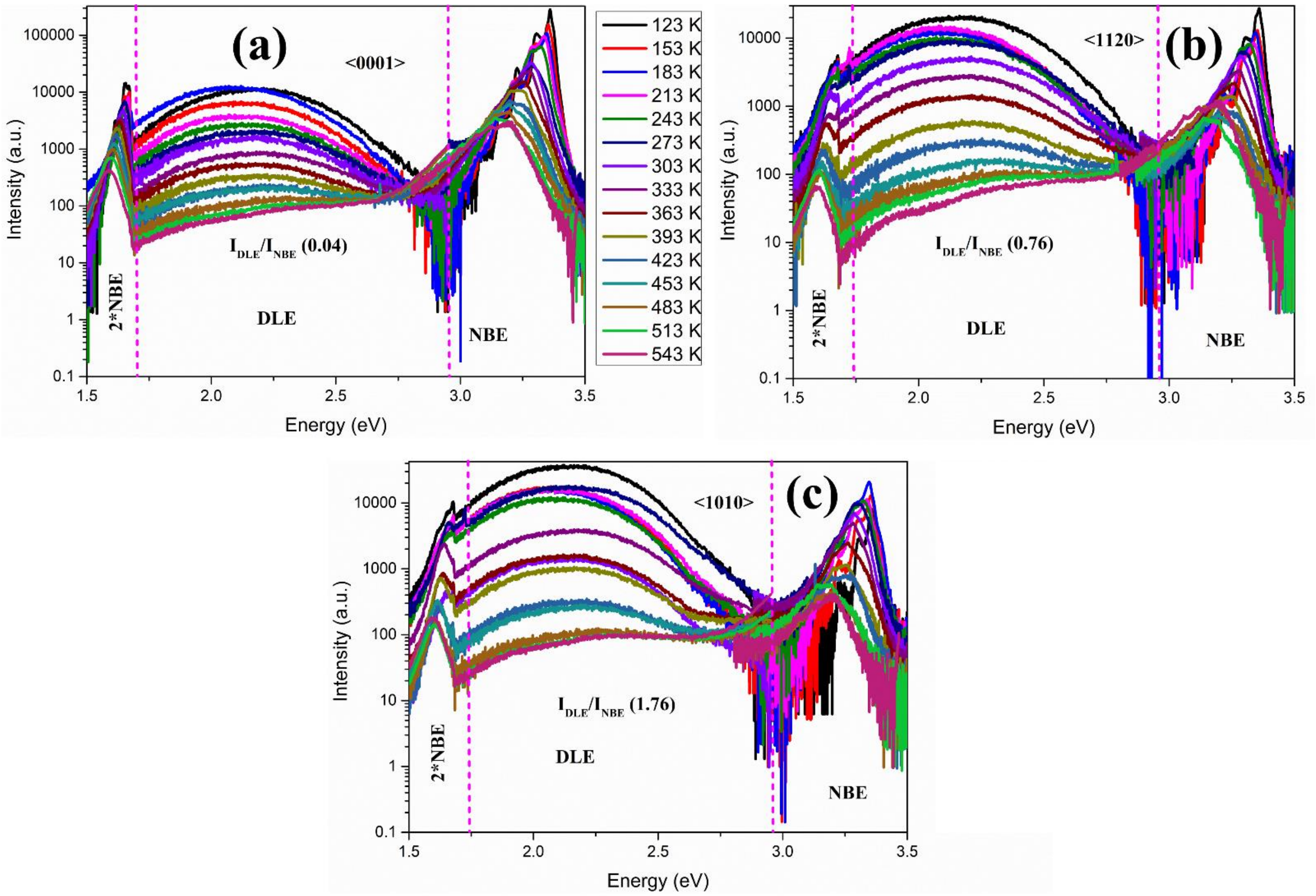

Figure S1: Complete scan of temperature-dependent photoluminescence spectra of (a) <0001> (b) <1120> (c) <1010> orientation of ZnO single crystals measured with 320 nm excitation wavelength over the temperature range of 123-543 K. Here NBE and DLE represents the near-band-edge and deep level emission, respectively. I_{NBE} and I_{DLE} denotes the intensity of NBE and DLE.

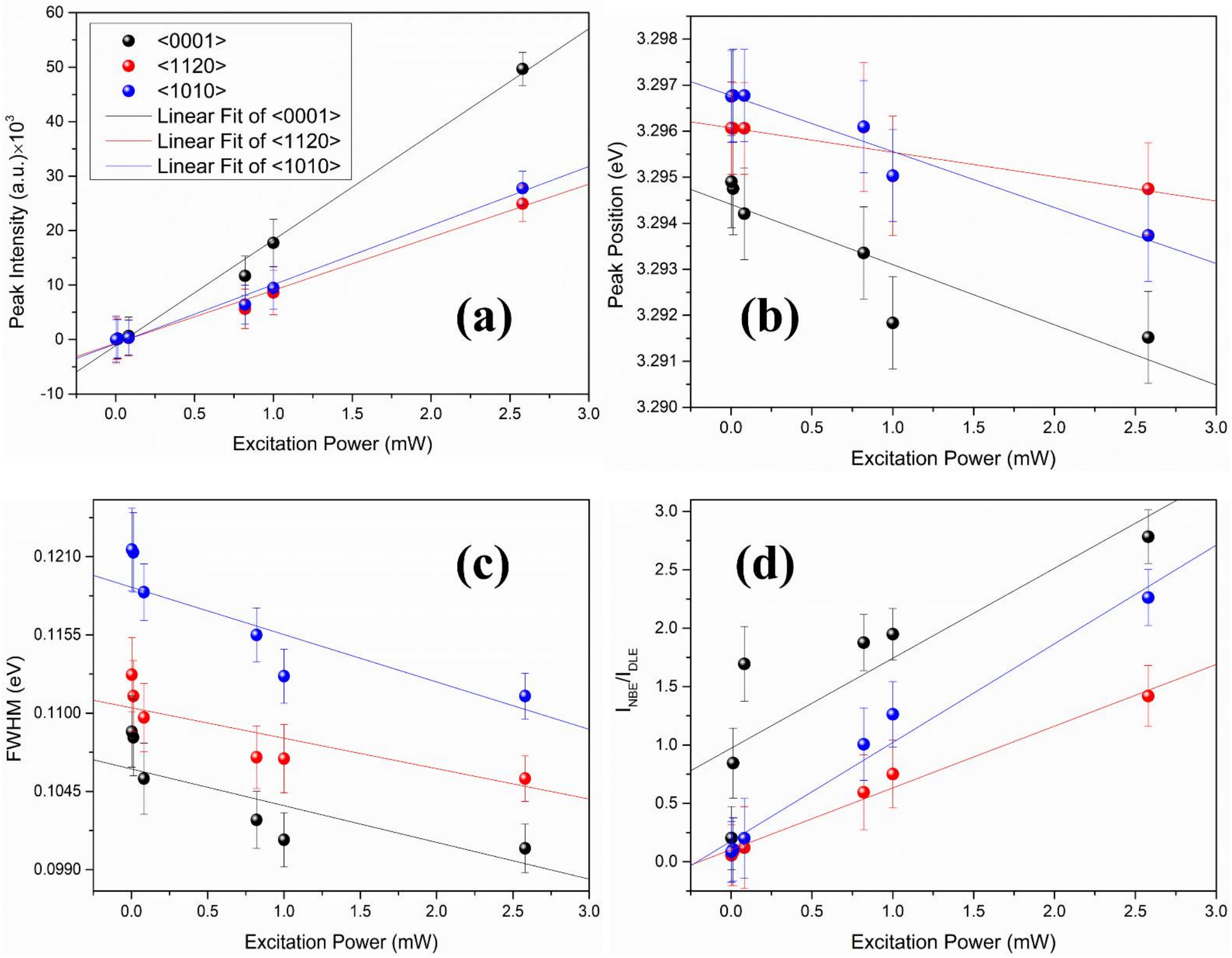

Figure S2: The relationship between (a) peak intensity (b) peak position (c) FWHM (d) ratio of NBE/DLE intensity as a function of excitation power for ZnO single crystals with three distinct orientations . The solid lines represent a linear fit of the experimentally observed data points, while the black, red, and blue solid spheres represent experimentally observed data of <0001>, <1120>, and <1010> ZnO single crystals.

Table S1: Room-temperature PL peak position, intensity ratio and the Varshni's fit parameters for ZnO single crystals with different orientations.

Orientation Type	NBE Peak Position (eV)	DLE Peak Position (eV)	I_{DLE}/I_{NBE}	α (eV/K)	β (K)
<0001>	3.28	2.17	0.074	0.00104	897
<1120>	3.29	2.15	1.434	0.00102	928
<1010>	3.29	2.15	1.705	0.00109	1016
